# Sodium-Coupled Neutral Amino Acid Transporter 1 (SNAT1) Modulates L-Citrulline Transport and Nitric Oxide (NO) Signaling in Piglet Pulmonary Arterial Endothelial Cells

**DOI:** 10.1371/journal.pone.0085730

**Published:** 2014-01-15

**Authors:** Anna Dikalova, Angela Fagiana, Judy L. Aschner, Michael Aschner, Marshall Summar, Candice D. Fike

**Affiliations:** 1 Dept. of Pediatrics, Vanderbilt University Medical Center, Nashville, Tennessee, United States of America; 2 Monroe Carell Jr. Children's Hospital at Vanderbilt, Nashville, Tennessee, United States of America; 3 Vanderbilt Center for Molecular Toxicology, Nashville, Tennessee, United States of America; 4 Division of Genetics and Metabolism, Children's National Medical Center, Washington, District of Columbia, United States of America; 5 Dept of Pediatrics, Albert Einstein College of Medicine and the Children's Hospital at Montefiore, New York, New York, United States of America; Indiana University, United States of America

## Abstract

**Rationale:**

There is evidence that impairments in nitric oxide (NO) signaling contribute to chronic hypoxia-induced pulmonary hypertension. The L-arginine-NO precursor, L-citrulline, has been shown to ameliorate pulmonary hypertension. Sodium-coupled neutral amino acid transporters (SNATs) are involved in the transport of L-citrulline into pulmonary arterial endothelial cells (PAECs). The functional link between the SNATs, L-citrulline, and NO signaling has not yet been explored.

**Objective:**

We tested the hypothesis that changes in SNAT1 expression and transport function regulate NO production by modulating eNOS coupling in newborn piglet PAECs.

**Methods and Results:**

A silencing RNA (siRNA) technique was used to assess the contribution of SNAT1 to NO production and eNOS coupling (eNOS dimer-to-monomer ratios) in PAECs from newborn piglets cultured under normoxic and hypoxic conditions in the presence and absence of L-citrulline. SNAT1 siRNA reduced basal NO production in normoxic PAECs and prevented L-citrulline-induced elevations in NO production in both normoxic and hypoxic PAECs. SNAT1 siRNA reduced basal eNOS dimer-to-monomer ratios in normoxic PAECs and prevented L-citrulline-induced increases in eNOS dimer-to-monomer ratios in hypoxic PAECs.

**Conclusions:**

SNAT1 mediated L-citrulline transport modulates eNOS coupling and thus regulates NO production in hypoxic PAECs from newborn piglets. Strategies that increase SNAT1-mediated transport and supply of L-citrulline may serve as novel therapeutic approaches to enhance NO production in patients with pulmonary vascular disease.

## Introduction

Infants with chronic cardiopulmonary disorders associated with persistent or episodic hypoxia develop pulmonary hypertension. Impairments in nitric oxide (NO) signaling may contribute to the development of chronic hypoxia-induced pulmonary hypertension [1], [2]. NO production from endothelial nitric oxide synthase (eNOS) is regulated in part by the availability of the substrate, arginine, and the cofactor, tetrahydrobiopterin (BH_4_) [3], [4], [5]. In the absence of sufficient arginine or BH_4_, eNOS activation generates superoxide (O_2_^•−^) instead of NO, a process known as NOS uncoupling [3], [4], [5]. Mechanisms that drive NOS re-coupling are poorly defined but provide potentially powerful therapeutic targets. Since L-arginine promotes eNOS coupling, strategies that effectively increase intracellular L-arginine availability to eNOS could prove beneficial. While there is evidence that direct L-arginine supplementation may be effective treatment in some experimental models of pulmonary hypertension [5], [6], [7] detrimental effects of L-arginine supplementation have also been reported and results from L-arginine treatment have been variable [8], [9], [10], [11]. Thus, alternate means for driving NOS re-coupling and increasing NO production merit further exploration.

The L-arginine-NO precursor, L-citrulline, provides an alternate approach to deliver bioavailable L-arginine for NO production. There is evidence that in endothelial cells, L-citrulline is converted by a two-step enzymatic process to L-arginine which is directly channeled to eNOS for efficient NO production [9], [12]. Surprisingly, little is known about the transport of L-citrulline into pulmonary arterial endothelial cells (PAECs). This knowledge could provide another means to manipulate NO production.

We recently showed that sodium-coupled neutral amino acid transporters (SNATs) are involved in transporting L-citrulline into PAECs under both normoxic and hypoxic conditions [13]. Expression of SNAT1 is increased in PAECs cultured under hypoxic conditions [13]. However, the link between SNAT1 expression, L-citrulline uptake, and NO signaling has not been explored. The major purpose of this study was to test the hypothesis that changes in SNAT1 expression and transport function regulate NO production by modulating eNOS coupling in newborn piglet PAECs.

## Methods

### Ethics statement

Use of animals conformed to the Guide for the Care and Use of Laboratory Animals published by the US National Institutes of Health (NIH Publication No. 85-23) and was approved by the Institutional Animal Care and Use Committee of Vanderbilt University Medical Center, which is fully accredited by the Association for Assessment and Accreditation of Laboratory Animal Use.

### PAEC isolation

Using previously published methods [13], the main pulmonary artery was isolated from the lungs of 5-day-old York-Landrace mixed breed piglets, flushed with PBS, then filled with 0.25% trypsin-EDTA and incubated for 5 min. To remove the endothelial cells, the pulmonary artery was gently flushed with endothelial growth medium (EGM-2, Lonza). Harvested endothelial cells were cultured in EGM-2 in 100 mm plates in a humidified, normoxic incubator (21% O_2_, 5% CO_2_) at 37°C. PAECs were identified by their cobblestone morphology and eNOS-positive staining. Cells were subcultured at near confluence and used at passages 4–10.

### Modulation of SNAT1 expression

Using a modification of methods previously described [14], PAECs were transfected with non-targeting (control) oligonucleotides (siGENOME Non-targeting siRNA #5) or SNAT1 targeting oligonucleotides (sense, 5′-ACGAACAGCCAUUUGGAAAUU-3′; anti-sense, 5′-UUUCCAAAUGGCUGUUCGUUU-3′; sense, 5′-CCGGAAGAUGAUAACAUUAUU-3′ anti-sense, 5′-UAAUGUUAUCAUCUUCCGGUU-3′; anti-sense, 5′-UAAUGUUAUCAUCUUCCGGUU-3′; sense, 5′-CAGUAACACUUCUGUCUAUUU-3′ anti-sense, 5′-AUAGACAGAAGUGUUACUGUU-3′) purchased from Thermo Scientific. For transfection, EGM-2 was replaced with OPTI-MEM (Gibco). PAECs were transfected with a 300–600 pmol suspension of either non-targeting or SNAT1 oligonucleotides (100–200 pmol for each of the 3 different SNAT1 targeting oligonucleotides) using lipofectamine (Invitrogen) in OPTI-MEM. After 4 hours, the medium was replaced with EGM-2 and 10% FBS.

### L-Citrulline uptake

After 48 hours in normoxic (21% O_2_, 5% CO_2_) or hypoxic (4% O_2_, 5% CO_2_) conditions, PAECs were prepared for measuring L-citrulline uptake using methods previously described [13]. PAEC were washed with 2 mL fresh sodium-HEPES buffer composed of the following: 2 mol/L NaCl, 2 mol/L KCl, 0.3 mol/L MgSO_4_, 0.325 mol/L CaCl_2_, 0.3 mol/L KH_2_PO_4_ and 1 mol/L HEPES adjusted to pH of 7.4 with 1 mol/L NaOH. Cells were incubated under normoxic conditions (21% O_2_, 5% CO_2_, 37°C) with 0.25 µCi/mL ^14^C-L-citrulline (specific activity: 56.3 mCi/mmol; Perkin Elmer, Norwalk, CT, USA) in the presence of a saturating concentration (200 µmol/L) of unlabeled L-citrulline. Incubations were stopped after 5–10 min by washing three times with 2 mL ice-cold sodium-HEPES buffer. All cells were lysed with 450 µL 1 mol/L NaOH for 30 min at 37°C. A 400 µL aliquot of each lysate was transferred to a scintillation vial, LSC cocktail (Fisher Scientific, Pittsburgh, PA, USA) was added and radioactivity was measured in a scintillation counter (Beckman LS 6500; Brea, CA, USA). The remainder of the lysate was used for protein determination by bicinchoninic acid protein assay (Pierce, Rockford, IL, USA). Uptake of L-citrulline was expressed as radioactive counts per minute (CPM) per mg of protein.

### NO measurement by ESR

PAECs were incubated under normoxic (21% O_2_, 5% CO_2_) or hypoxic (4% O_2_, 5% CO_2_) conditions for 48 hours in basal media (EGM-2), which contains 4 µmol/L L-citrulline. During the final hour of the 48-hour incubation period, L-citrulline was added to the medium of some cells to achieve a final conc. of 1 mmol/L. Cells were washed with chilled Krebs-HEPES buffer and incubated with calcium ionophore, A-23187 (10 µmol/L) and 200 µmol/L Fe(DETC)_2_ at 37°C for 1 hour. The cells were then scraped in 0.5 ml of Krebs-HEPES buffer and snap-frozen in liquid nitrogen and stored at −80°C until assessed for NO production by electron spin resonance (ESR) as previously described [14]. The amount of detected NO was determined from the calibration curve for integral intensity of the ESR signal of NO-Fe^2+^(MGD)_2_ prepared at various concentrations of the NO-donor MAHMA-NONOate (ENZO Life Sciences Inc. NY, USA).

### Superoxide measurement using dihydroethidium and an HPLC-based assay

Superoxide was measured using dihdroethidium (DHE) and an HPLC-based assay as previously described [14]. PAECs were cultured under normoxic or hypoxic conditions for 48 hours in basal media (EGM-2), which contains 4 µmol/L L-citrulline. During the final hour of the 48-hour incubation period, L-citrulline was added to the medium of some cells to achieve a final conc. of 1 mmol/L. PAECs were washed three times with chilled Krebs-HEPES buffer and then incubated with 25 µmol/L dihydroethidium for 20 min at 37°C in Krebs-HEPES buffer. The cells were harvested in 0.3 ml of methanol, homogenized and filtered through 0.22 µm filters and stored at −80°C until analyzed by HPLC. Protein measurements by Bradford assay were done in aliquots of homogenates collected prior to filtration. Separation of ethidium, 2-hydroxyethidium, and dihydroethidium was performed using a Beckman HPLC System Gold model with a C-18 reverse phase column (Nucleosil 250, 4.5 mm; Sigma-Aldrich, St. Louis, MO) equipped with both UV and fluorescence detectors. Fluorescence detection at 580 nm (emission) and 480 nm (excitation) was used to monitor 2-hydroxyethidium production. UV absorption at 355 nm was used for the detection of dihydroethidium. The mobile phase was composed of a gradient containing 60% acetonitrile and 0.1% trifluoroacetic acid. Dihydroethidium, ethidium and 2-hydryoxyethidium were separated by a linear increase in acetonitrile concentration from 37 to 47% over 23 min at a flow rate of 0.5 mL/min. Hydroxyethidium was expressed per milligram protein.

### Immunoblot analysis of SNAT1, eNOS, and eNOS dimers and monomers

PAECs were washed with PBS, then collected and stored at −80°C. Frozen PAECs were crushed under liquid N_2_ into a fine powder, transferred to a tube containing homogenization buffer with protease inhibitors, and then sonicated. Protein concentrations for all homogenates were determined by the protein assay (Bradford). For SNAT1 and eNOS analysis, using previously described methods [13], supernatants were applied to tris-glycine pre-cast 4–20% polyacrylamide gels (Invitrogen, Carlsbad, CA, USA) so that equal amounts of protein were loaded. Electrophoresis was carried out and the proteins were transferred from the gel to a nitrocellulose membrane. The membrane was incubated at room temperature in PBS containing 7.5% non-fat dried milk and 0.1% Tween-20 to block non-specific protein binding. To detect SNAT1 or eNOS, the nitrocellulose membrane was incubated overnight with the primary antibody (SNAT1 1∶800, ABCAM; eNOS 1∶2000, eNOS antibody from BD-Transduction Laboratory, San Diego, CA) diluted in PBS containing 0.1% Tween-20 and 1% non-fat dried milk (carrier buffer); followed by incubation with a horseradish peroxidase-conjugated secondary antibody (Zymed) diluted in the carrier buffer (1∶5000). Using non-sonicated and nonboiled lysates and low-temperature SDS-PAGE, eNOS dimers/monomers were immunoblotted (1∶2000, eNOS antibody, BD-Transduction Laboratory, San Diego, CA) as described elsewhere [15]. The membranes were developed using enhanced chemiluminescence reagents (ECL, Amersham) and the chemiluminescent signal was captured on X-ray film (ECL Hyperfilm, Kodak). Similar procedures were followed to reprobe the membranes for β-actin (Sigma-Aldrich, St. Louis, MO) or GAPDH (ABCAM, 1∶1000). The bands for each protein were quantified using densitometry.

### Statistical Analysis

Data are presented as mean ± SEM. Data were compared by unpaired t-test or one-way ANOVA with Fisher's protected least significant difference (PLSD) post hoc comparison test as appropriate. P-values <0.05 were considered significant.

## Results

**Figure 1:**
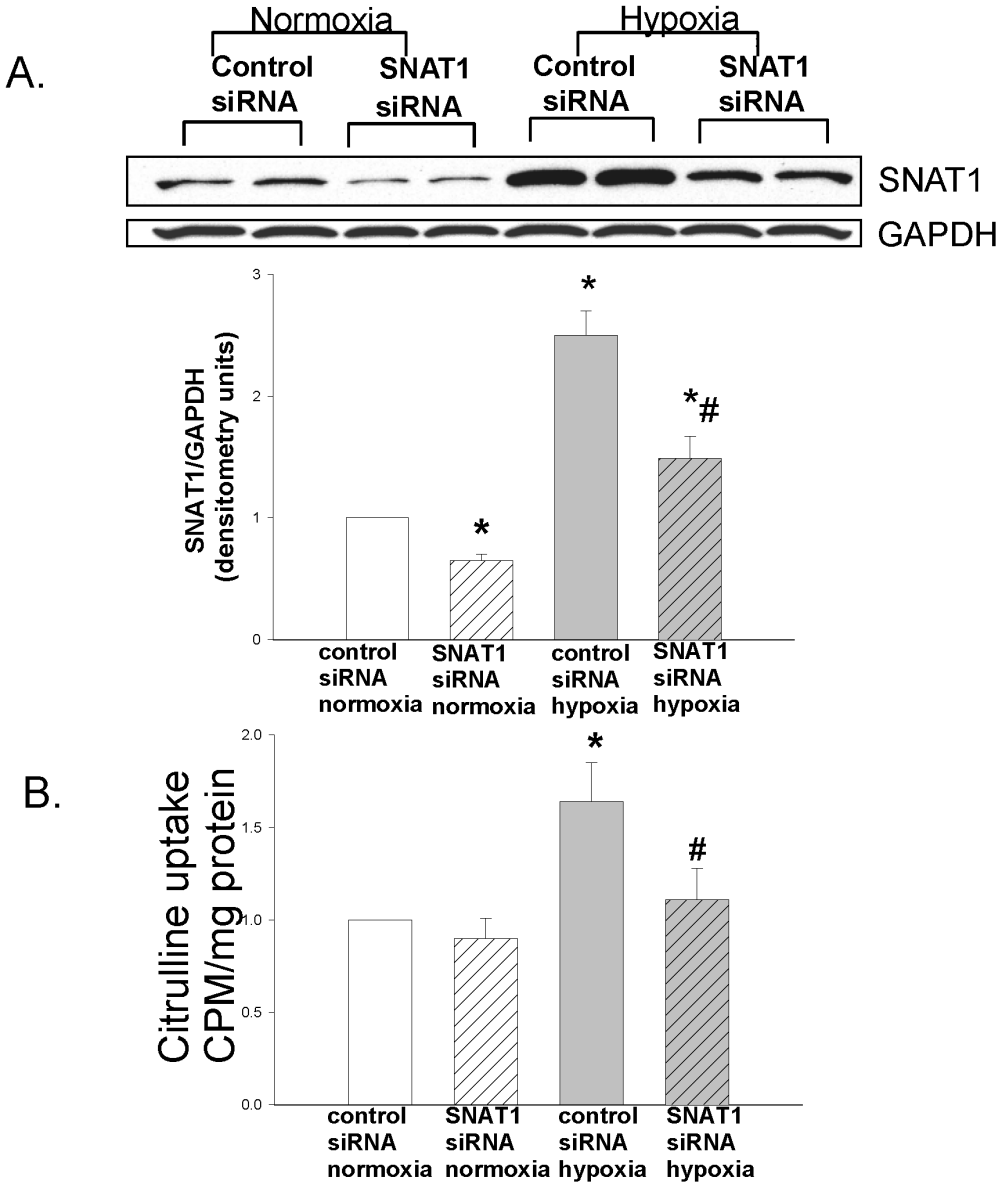
SNAT1 expression and L-citrulline uptake in PAECs cultured under normoxic or hypoxic conditions. Hypoxia increased both the expression of the amino acid transporter, SNAT1 (A), and the uptake of ^14^C-L-citrulline (B) in PAECs (n = 11) from newborn piglets. In normoxic PAECs (n = 11), SNAT1 siRNA reduced SNAT1 expression (A) without having a detectable impact on ^14^C-L-citrulline uptake (B: L-citrulline uptake in normoxic PAECs treated with SNAT1 siRNA was 0.9±0.11; p = 0.4). *different from normoxia control siRNA, #different from hypoxia control siRNA; P<0.05.

Hypoxia increased SNAT1 expression (p<0.001; Fig. 1A) and L-citrulline uptake (p = .003; Fig. 1B) in PAECs. In normoxic PAECs, SNAT1 siRNA reduced SNAT1 expression (p = 0.04; Fig. 1A) without having a detectable impact on L-citrulline uptake (p = 0.4; Fig. 1 B). More importantly, SNAT1 siRNA inhibited the hypoxia-induced elevation in SNAT1 expression (p<0.001; Fig. 1A) and concomitantly prevented the hypoxia-induced increase in L-citrulline uptake (p = 0.01; Fig. 1B). These findings indicate that hypoxia increases L-citrulline uptake in PAECs via SNAT1 and demonstrate an association between SNAT1 and the transport of the L-arginine-NO substrate, L-citrulline, in PAECs of newborn piglets.

**Figure 2:**
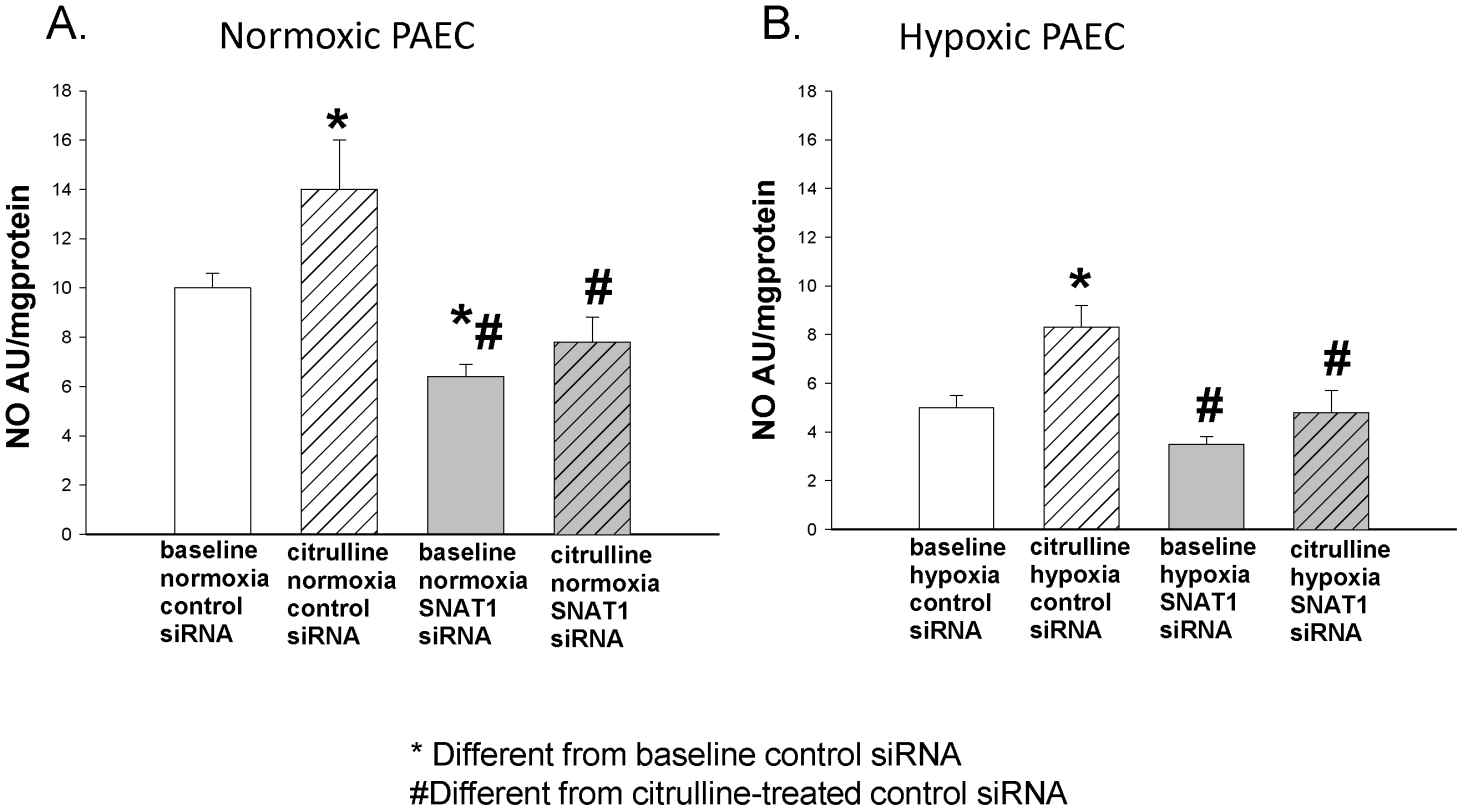
NO production in PAECs cultured under normoxic or hypoxic conditions. SNAT1 siRNA prevented L-citrulline-induced increases in NO production in both normoxic (A, n = 5) and hypoxic (B, n = 5) PAECs from newborn piglets. A: for SNAT1 siRNA treated normoxic PAECs in the absence *vs* presence of L-citrulline NO production was respectively, 6.4±0.5 and 7.8±1.0 AU/mg protein, p = 0.4. B: for SNAT1 siRNA treated hypoxic PAECs in the absence *vs* presence of L-citrulline, NO production was respectively, 3.5±0.3 and 4.8±0.9 AU/mg protein, p = 0.21. *different from baseline control siRNA, #different from citrulline-treated control siRNA, P<0.05.

To explore the role of L-citrulline and SNAT1 in modulating NO signaling in PAECs, we determined the effects of L-citrulline treatment and SNAT1 depletion by siRNA on NO production in normoxic and hypoxic PAECs. L-citrulline significantly increased NO production in both normoxic (p = 0.03; Fig. 2A) and hypoxic (p = 0.005; Fig. 2B) PAECs. SNAT1 siRNA prevented the L-citrulline-induced increase in NO production in both normoxic (Fig. 2A; p = 0.4) and hypoxic PAECs (Fig. 2B, p = 0.21). These findings show that L-citrulline-induced NO production is modulated by SNAT1.

**Figure 3:**
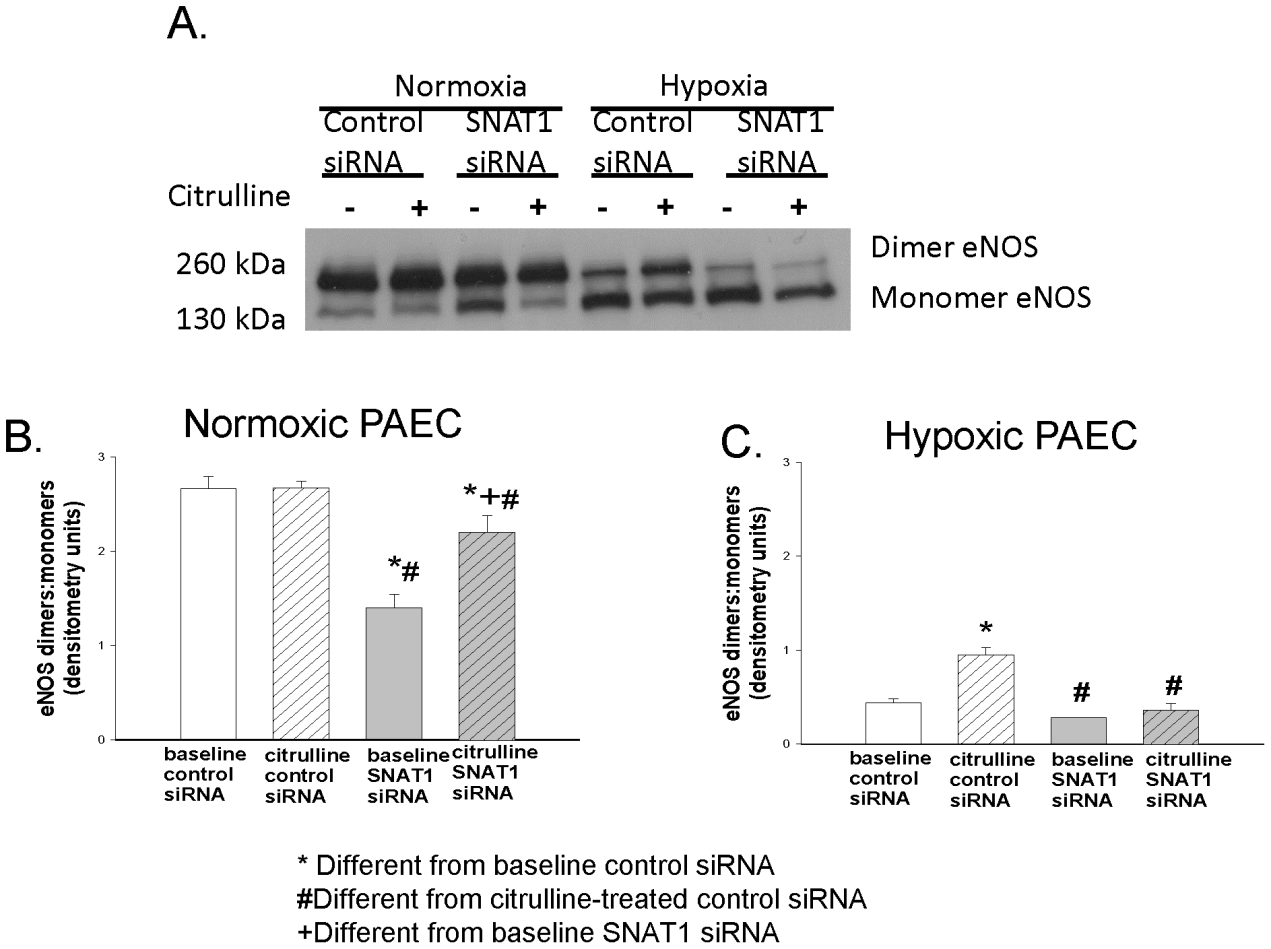
eNOS dimers/monomers in PAECs cultured under normoxic or hypoxic conditions. Representative western blot (A); densitometry for normoxic (B, n = 5) and hypoxic (C, n = 5) PAECs. In normoxic PAECs, L-citrulline had no effect on the eNOS dimer-to-monomer ratio when transfected with control siRNA (A and B: in the absence *vs* presence of L-citrulline eNOS dimer-to-monomer ratio was respectively: 2.7±0.13 and 2.7±0.07, p = 0.97) but increased the eNOS dimer-to-monomer ratio when transfected with SNAT1 siRNA. In hypoxic PAECs, L-citrulline increased eNOS dimer-to-monomer ratios when transfected with control siRNA but did not increase the eNOS dimer-to-monomer ratio when transfected with SNAT1 siRNA (A and C: in the absence vs presence of L-citrulline, eNOS dimer-to-monomer ratio was respectively: 0.28±0.003 vs 0.37±0.07, p = 0.3). *different from baseline control siRNA, #different from L-citrulline-treated control siRNA, +different from baseline SNAT1 siRNA; P<0.05.

**Figure 4:**
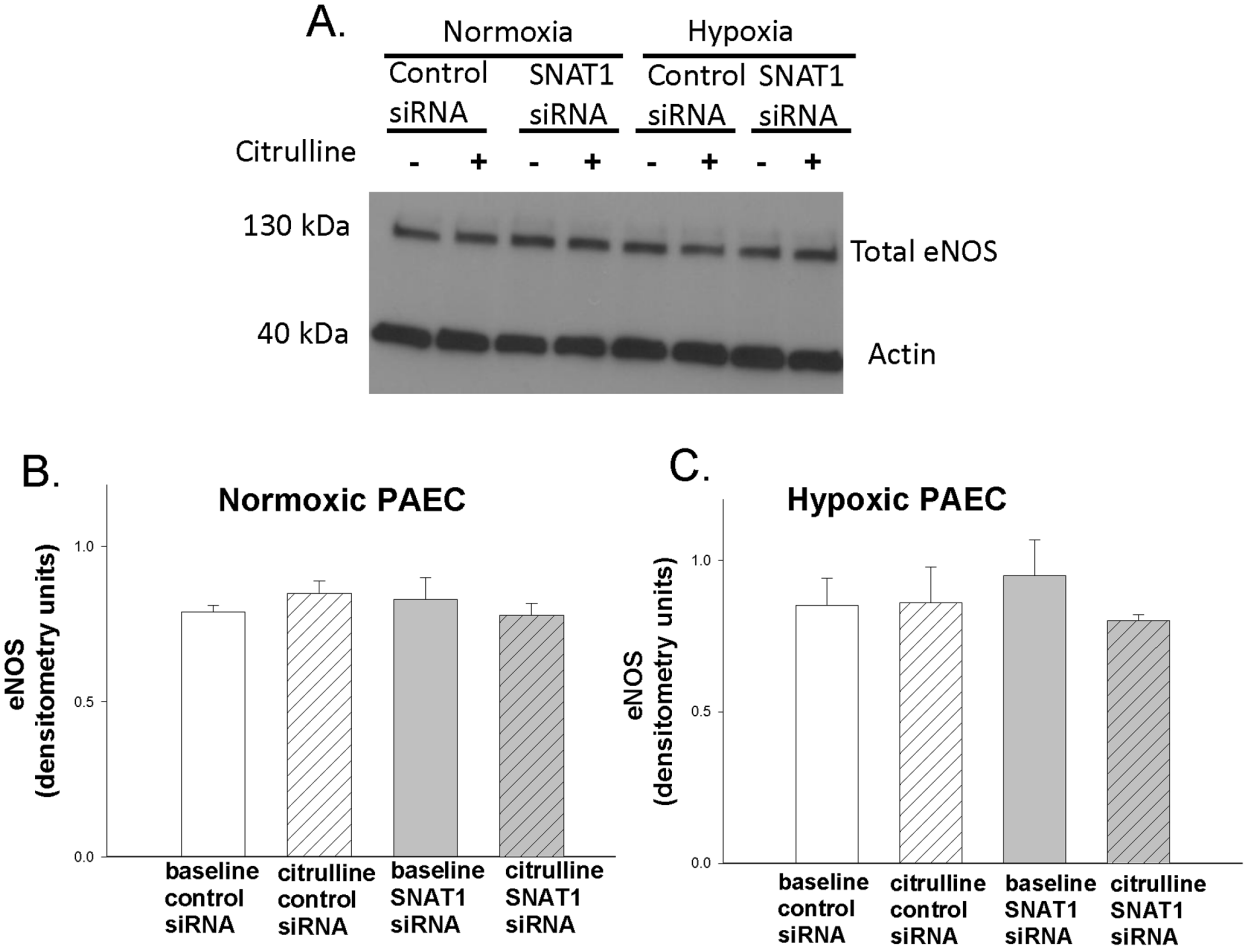
Total eNOS measured under baseline conditions and with L-citrulline treatment in PAECs cultured under normoxic or hypoxic conditions. A: representative western blot: B: densitometry for normoxic PAECs, n = 5; C: densitometry for hypoxic PAECs, n = 5. Neither L-citrulline nor SNAT1 siRNA altered total eNOS expression in either normoxic or hypoxic PAECs.

NO production is influenced by the state of eNOS coupling. Uncoupled eNOS can be demonstrated as a loss of eNOS dimer formation and an increase of eNOS monomers. We determined the effects of L-citrulline treatment and SNAT1 siRNA on eNOS dimer-to-monomer ratios in normoxic and hypoxic PAECs. L-citrulline had no effect on the eNOS dimer-to-monomer ratio in normoxic PAECs transfected with control siRNA (Fig. 3A and 3B; p = 0.97). L-citrulline increased the eNOS dimer-to-monomer ratio in normoxic PAECs transfected with SNAT1 siRNA (Fig. 3A and 3B; p = 0.001). SNAT1 siRNA decreased both baseline eNOS dimer-to-monomer ratios (Fig. 3A and 3B; p<0.001) and baseline NO production (Fig. 2A; p = 0.03) in normoxic PAECs. Neither L-citrulline nor SNAT1 siRNA altered total eNOS expression in normoxic PAECs (Figure 4; p = 0.7). These data suggest that SNAT1 knockdown reduced baseline NO production in normoxic PAECs, at least in part, by uncoupling eNOS.

In hypoxic PAECs, L-citrulline increased eNOS dimer-to-monomer ratios (Fig. 3A and 3C; p = 0.002), an effect that was abolished by SNAT1 siRNA (Fig. 3A and 3C; p = 0.3). Total eNOS expression was unchanged by L-citrulline and SNAT1 siRNA (Figure 4; p = 0.7). In conjunction with the NO data in Figure 2 B, these findings suggest that L-citrulline and SNAT1 modulate NO production in hypoxic PAECs by influencing the state of eNOS coupling.

**Figure 5:**
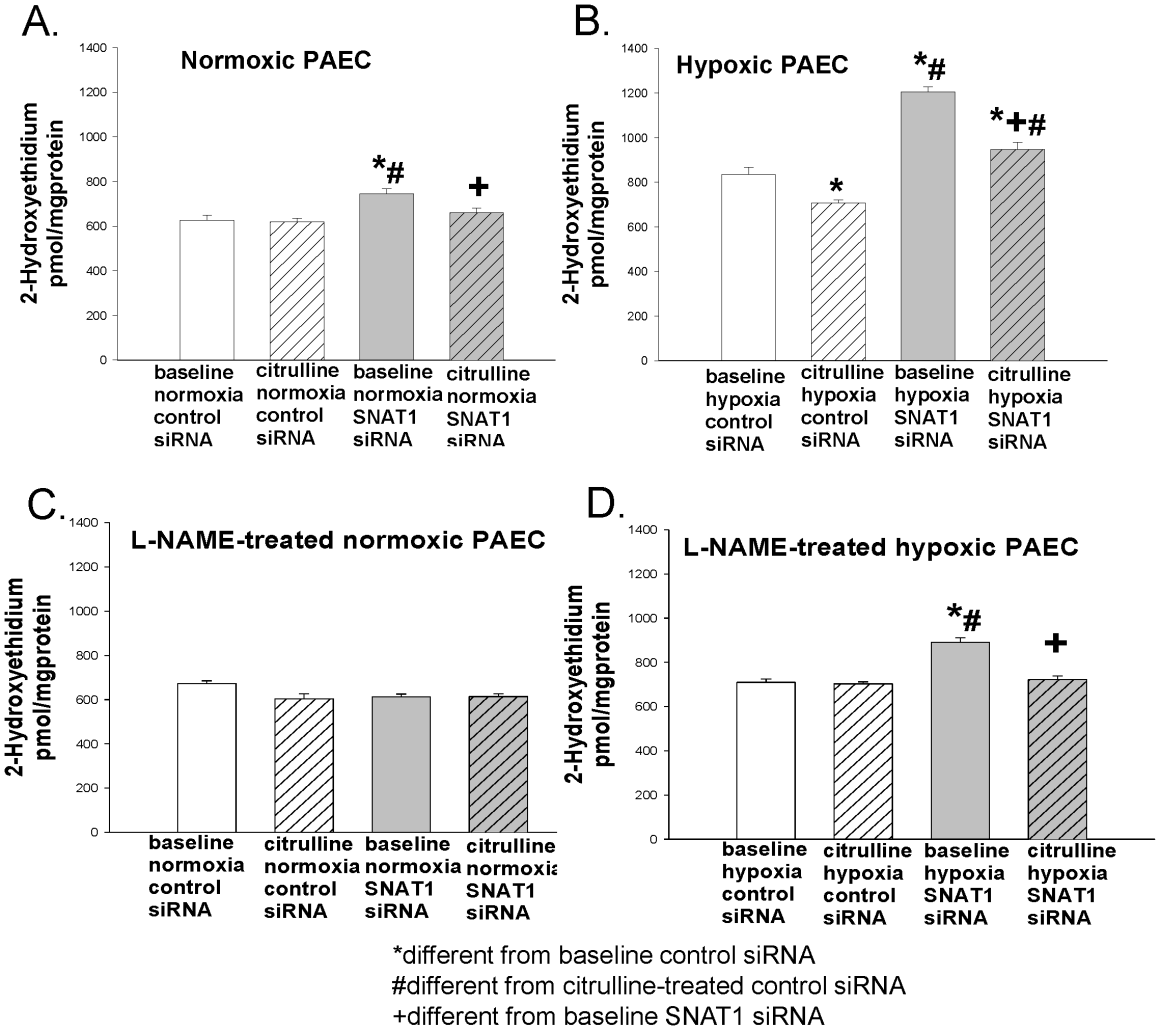
Superoxide production, as assessed by the formation of 2-hydroxyethidium, in PAECs cultured under normoxic or hypoxic conditions. Normoxic PAECs (n = 5) in the absence (A) or presence (C) of L-NAME; Hypoxic PAECs (n = 5) in the absence (B) or presence (D) of L-NAME. In normoxic conditions, L-citrulline had no effect on superoxide production in PAECs transfected with control siRNA in either the presence (C) or absence (A) of L-NAME (A: O_2_^•−^ production in the absence *vs* presence of L-citrulline was respectively, 625±23 and 603±28 pmol/mg protein, p = 0.5 and C: O_2_^•−^ production in the absence vs presence of L-citrulline was respectively, 670±12 and 610±28 pmol/mg protein, p = 0.08). L-citrulline reduced superoxide production in PAECs transfected with SNAT1 siRNA in the absence (A) but not the presence (C) of L-NAME (C: O_2_^•−^ production in the absence *vs* presence of L-citrulline was respectively, 613±12 and 614±13 pmol/mg protein, p = 0.9). Moreover, SNAT1 siRNA increased baseline O_2_^•−^ production in normoxic PAEC in the absence (A) but not the presence (C) of L-NAME (C: O_2_^•−^ production for control siRNA *vs* SNAT1 siRNA was respectively, 670±12 and 613±12 pmol/mg protein, p = 0.08). In hypoxic conditions, L-citrulline reduced superoxide production in PAECs transfected with control siRNA in the absence (B) but not the presence of L-NAME (D: O_2_^•−^ production in the absence *vs* presence of L-citrulline was respectively, 711±14 and 703±9 pmol/mg protein, p = 0.8). L-citrulline reduced superoxide production in PAECs transfected with SNAT1 siRNA in both the presence (D) and the absence (B) of L-NAME. *different from baseline control siRNA; #different from L-citrulline-treated control siRNA; +different from baseline SNAT1 siRNA; P<0.05.

We also determined the effects of L-citrulline treatment and SNAT1 siRNA on O_2_^•−^ production in normoxic and hypoxic PAECs in the absence and presence of the NOS inhibitor, L-NAME. L-citrulline had no effect on O_2_^•−^ production in normoxic PAECs transfected with control siRNA in either the presence or absence of L-NAME, (Fig. 5A, p = 0.5 and 5C, p = 0.08). In contrast, L-citrulline reduced O_2_^•−^ production in normoxic PAECs transfected with SNAT1 siRNA in the absence of L-NAME (Fig. 5A, p = 0.036) but not the presence of L-NAME (Fig. 5C, p = 0.9). Moreover, SNAT1 siRNA increased baseline O_2_^•−^ production in normoxic PAEC in the absence of L-NAME (Fig. 5A; p = 0.008) but not the presence of L-NAME (Fig. 5C, p = 0.08). That is, in the presence of L-NAME (Fig. 5C), neither L-citrulline nor SNAT1 siRNA caused changes in O_2_^•−^ production in normoxic PAECs. In addition, changes in O_2_^•−^ production in normoxic PAEC in the absence of L-NAME (Fig. 5A) were concordant with changes in eNOS dimer-to-monomer ratios (Fig. 3A and 3B). Taken together, these findings show that both L-citrulline and SNAT1 modulate O_2_^•−^ production in normoxic PAECs in a NOS-dependent fashion that reflects the state of eNOS coupling.

In the absence of L-NAME (Fig. 5 B), L-citrulline reduced O_2_^•−^ production in hypoxic PAECs transfected with either control siRNA (p = 0.01) or SNAT1 siRNA (p = 0.001). In the presence of L-NAME, L-citrulline had no effect on O_2_^•−^ production in hypoxic PAECs transfected with control siRNA (Fig. 5D, p = 0.8). However, in the presence of L-NAME, reductions in O_2_^•−^ production with L-citrulline persisted in hypoxic PAECs transfected with SNAT1 siRNA (p = 0.001; Fig. 5D). An increase in baseline O_2_^•−^ production occurred with SNAT1 siRNA in hypoxic PAECs both in the absence (p = <0.001; Fig. 5B) and presence (p = <0.001; Fig. 5D) of L-NAME. In the latter case, the magnitude of increase in O_2_^•−^ production was reduced by the presence of L-NAME (increase in O_2_^•−^ production was 44±4% *vs* 25±5% respectively in the absence *vs* the presence of L-NAME, p<0.05). These findings show that both L-citrulline and SNAT1 modulate O_2_^•−^ production in hypoxic PAECs in a NOS-dependent fashion.

## Discussion

Our studies reveal a number of novel findings regarding L-citrulline and NO signaling in PAECs. We show for the first time that the neutral amino acid transporter, SNAT1, modulates L-citrulline-induced increases in NO production. Moreover, we provide important new evidence that L-citrulline supplementation increases NO production by re-coupling eNOS in a SNAT1-dependent manner in hypoxic PAECs.

There are limited data on the transport of the amino acid, L-citrulline, by neutral amino acid transporters in vascular cells [16]. Information about the impact of hypoxia on neutral amino acid transporters, including SNAT1, in cells from vascular beds is also scarce [17]. L-citrulline uptake was reported to remain unchanged in PAECs from adult pigs cultured for up to 24 hours in hypoxia [18]. However, we previously found that L-citrulline uptake was increased in PAECs from newborn piglets cultured under hypoxic conditions for 24, 48, or 72 hours [13]. Moreover, we provided evidence that the hypoxia-induced increase in L-citrulline transport involved the System A family of sodium-coupled neutral amino acid transporters [13]. We confirm our previous findings and extend them by identifying SNAT1 as the System A transporter that is responsible for the enhanced ability to transport L-citrulline in hypoxic PAECs.

To our knowledge, we are the first to provide evidence of an important functional link between SNAT1, L-citrulline, and NO signaling in PAECs. Others have shown that L-citrulline can increase NO production in endothelial cells [19]. However, our findings provide the first mechanistic evidence that SNAT1 is integral to the effect of L-citrulline on NO production in both normoxic and hypoxic PAECs. Furthermore, we are the first to show that SNAT1 is involved in modulating eNOS uncoupling and O_2_^•−^ production in PAECs.

The role of SNAT1 in modulating eNOS re-coupling and O_2_^•−^ production. differs between normoxic and hypoxic PAECs. SNAT1 siRNA reduced baseline eNOS dimer formation and also increased O_2_^•−^ production in normoxic PAECs indicating that SNAT1 is essential for either maintaining or promoting eNOS dimerization under basal conditions, thereby impacting basal O_2_^•−^ and NO production. However, despite transfection with SNAT1 siRNA, L-citrulline increased eNOS dimer-to-monomer ratios in normoxic PAECs, suggesting that other transporters are involved in the mechanism by which L-citrulline re-couples eNOS in normoxic PAECs. In contrast, SNAT1 is integral to the ability of L-citrulline to re-couple eNOS in hypoxic PAECs.

Findings in this study reveal additional novel information on the mechanisms underlying L-citrulline-induced increases in NO production. We have shown previously in an *in vivo* model that L-citrulline supplementation increases pulmonary vascular NO production and attenuates chronic hypoxia-induced pulmonary hypertension in newborn piglets [20]. Findings herein provide additional insight into the mechanisms by which L-citrulline might alleviate pulmonary hypertension. We show that when hypoxic PAECs are treated with L-citrulline there is a significant increase in the eNOS dimer∶monomer ratio, indicating that L-citrulline reduces eNOS uncoupling. Consistent with eNOS re-coupling, O_2_^•−^ generation is reduced and NO production is increased in hypoxic PAECs treated with L-citrulline. In addition, we demonstrate that the decrease in O_2_^•−^ seen with L-citrulline supplementation is abolished by treatment with L-NAME, further supporting that L-citrulline works in a NOS-dependent fashion.

Augmentation of NO production in normoxic PAECs occurs without a change in the state of eNOS coupling. Under normoxic conditions, eNOS dimers predominate making it biologically unlikely and technically difficult to demonstrate that greater eNOS coupling is driving enhanced NO production. The likely explanation for the increase in NO production with L-citrulline in normoxic PAECs is that, once transported into PAECs by SNAT1, L-citrulline is enzymatically converted to L-arginine by argininosuccinate synthetase (ASS) and argininosuccinate lyase (ASL), increasing the intracellular pool of L-arginine that is available to eNOS [9]. There is evidence that ASS-ASL-eNOS are co-localized in plasmalemmal caveolae of endothelial cells [19]. Hence, instead of equilibrating with bulk intracellular levels, L-citrulline induced increases in L-arginine could be directly channeled to eNOS thereby driving NO production [12].

Our study has some limitations that merit discussion. All studies were performed with PAECs isolated from the main pulmonary artery of newborn piglets. Whether our findings reflect endothelial cells from other species, other postnatal ages, all segments of the pulmonary circulation, or other vascular beds will require future exploration. We were unable to detect a significant reduction in L-citrulline uptake in normoxic PAECs treated with SNAT1 siRNA. Given the overlap of substrate specificity of amino acid transporters, SNAT1 would not be expected to be the only L-citrulline transporter. The presence of other transporters, which could compensate for SNAT1 knockdown, likely led to an undetectable net change in L-citrulline uptake in normoxic PAECs.

In summary, we provide novel, mechanistic data supporting an integral role for SNAT1 in modulating L-citrulline-induced changes in NO signaling in PAECs of newborn piglets. Our findings have potential therapeutic significance and support a role for L-citrulline supplementation as a means to reduce O_2_^•−^ generation and increase NO production thereby ameliorating the development of pulmonary hypertension in newborns. In addition, our findings provide the impetus for future research to investigate the possibility that manipulating L-citrulline transport could be a viable therapeutic approach to modulate NO production in a variety of vascular diseases.
